# Impact of Endogenic and Exogenic Oxidative Stress Triggers on Pregnant Woman, Fetus, and Child

**DOI:** 10.3390/ijms24065958

**Published:** 2023-03-22

**Authors:** Joško Osredkar

**Affiliations:** 1 Clinical Institute of Clinical Chemistry and Biochemistry, University Medical Centre Ljubljana, Zaloška c. 2., 1000 Ljubljana, Slovenia; josko.osredkar@kclj.si; 2 Faculty of Pharmacy, University of Ljubljana, Aškerčeva 7, 1000 Ljubljana, Slovenia

In all living organisms, there is a delicate balance between oxidation caused by reactive species (RS, also called free radicals) and antioxidant defence. A shift in this balance is due to a condition known as “oxidative stress” (OS), which can cause cellular damage that eventually leads to premature aging and many diseases. Oxidative stress is not actually a “disease” and does have a specific clinical diagnosis; instead it hides behind the symptoms and signs of the underlying disease. In other words, we can only measure it if we perform certain biochemical tests. These tests can be set for different biological samples.

Pregnancy is a dynamic process during which systemic and local changes occur in the mother. All these changes are necessary for the normal development of the foetus. Disorders in this process can lead to complications in pregnancy, changes in the growth path of the foetus, premature birth, and some other conditions (e.g., treatment of preeclampsia and gestational diabetes or the need for caesarean section), intrauterine growth restriction (IUGR), and small or large infants for gestational age.

Homeostasis of the maternal environment is maintained through a variety of mediators, including hormones, cytokines, oxidative status, and diet. Factors that interfere with this homeostasis can be endogenous or exogenous and are inflammation, oxidative stress, exposure to chemical agents, and lack or excess of food, which can jeopardize the growth and development of the foetus. During pregnancy, there is an increase in oxidative stress, a phenomenon caused by the usual systemic inflammatory response, resulting in a large amount of reactive oxygen species in the circulation. The development and maturation of the placenta is a complex process that requires coordinated regulation of trophoblast invasion and its differentiation and spread in the maternal decidua.

Special Issue entitled “Impact of Endogenic and Exogenic Oxidative Stress Triggers on Pregnant Woman, Fetus, and Child” of the International Journal of Molecular Sciences includes a total of 8 contributions: 4 Reviews and 4 Original Articles providing new information about the Endogenic and Exogenic Oxidative Stress Triggers and oxidative stress in pathological pregnancies and its impact on mothers and new-borns.

We are increasingly aware that the discovery of new biomarkers in biological fluids is one of the still unsolved challenges. Researchers face this challenge for several reasons, most notably the high complexity of the molecules present and their wide and dynamic range of concentrations in the bloodstream.

Jeličić Lj. et al. [1] extensively reviewed maternal distress during pregnancy and postpartum period. The review is organized and presented to explore and describe the effects of anxiety, stress, and depression in pregnancy and the postpartum period on adverse child developmental outcomes. The neurobiology of maternal distress and the transmission mechanisms at the molecular level to the foetus and child are noted. In addition, the paper discusses the findings of longitudinal studies in which early child development is monitored concerning the presence of maternal distress in pregnancy and the postpartum period.

During prenatal, but especially during perinatal and infant development, the brain adapts in response to a wide range of early experiences, which underlie the creation of developmental pathways, i.e., the development of language, cognitive skills, and socio-emotional competencies. These periods are of significant vulnerability and may be influenced by internal and external risk factors that affect foetal and infant development. The important interplay between genes and the environment during the prenatal and early postnatal period is presented. There is a complex interplay between specific models of developmental stages and potential risks. Among these, maternal mental illness is considered a developmental risk in early child development, so maternal stress during pregnancy should be considered as an important risk factor that may affect foetal and child development and behaviour. It can manifest itself as generalised stress, anxiety and depressive symptoms and is very common, as a significant number of women experience psychological distress during pregnancy and after childbirth.

When authors discuss the Factors and Biological Mechanisms Underlying the Transmission of Maternal Distress to the (Unborn) Child, they present a broad spectrum of signals originating from maternal stress experiences and may have a long-lasting effect on the child.

Authors conclude that most studies point to the association between maternal distress and offspring development, and that further clarification on the different contributions of maternal exposure is needed, including the nature of the exposure, the timing of exposure in pregnancy, the sex of the foetus, the nature of maternal symptoms, nutrition, exposure to toxins, delivery factors, medication effects, socio-demographic variables, and other potential mediators.

Maternal psychological factors contribute significantly to pregnancy complications and the adverse development of the (unborn) child. Maternal distress should be monitored prenatally and in the postnatal period in relation to specific mechanisms of biological transmission.

There is a need for further interdisciplinary research on the link between maternal mental disorders and foetal/child health and development, in particular on the biological mechanisms underlying the transmission of maternal distress to the (unborn) child, in order to achieve positive developmental outcomes and improve maternal and child well-being.

Socha MW et al. [2] in their review present the involvement of reactive oxygen species (ROS) in the cervical ripening process, emphasizing the molecular and biochemical pathways and the clinical implications. In the first part, they present oxidative stress and apoptosis. The link between oxidative stress and apoptosis has been demonstrated in three main pathways, the mitochondrial, endoplasmic and extrinsic pathways. ROS can directly and indirectly stimulate each of these pathways. The mitochondrial pathway is regulated by the permeability of the inner mitochondrial membrane, which is physiologically impermeable, and the permeability of the inner membrane is increased in the presence of excessive ROS. The endoplasmic reticulum pathway is another pathway that is significantly influenced by reactive oxygen species. The connection with calcium ions is important. Calcium ions are important for the proper functioning of mitochondria. Excess accumulation of mitochondrial Ca2+ can increase the permeability of the mitochondrial membrane, leading to its swelling and subsequently causing rupture, which results in the release of cytochrome c into the cytosol. Extremely important pathway of apoptosis activation is the extrinsic pathway mediated by the tumour necrosis factor receptors located in the cell membrane.

In the second part, they present Oxidative Stress and Inflammation in Cervical Tissue. Many of the mediators and enzymes involved in the regulation of the acute inflammatory response are also strongly involved in the regulation of the cervical ripening process. During cervical ripening, local vasodilatation occurs, resulting in increased vascular permeability, which in turn leads to an increased volume of water and an influx of inflammatory cells which secrets proinflammatory cytokines. Secreted ROS strongly influence the development of inflammation.

In the third part they present Reactive Nitrogen Species and Cervical Ripening. NO, as the main representative of RNS, can act as an intracellular messenger, a paracrine mediator and a neurotransmitter that directly and indirectly influences target tissues. The direct effect on target tissues is manifested by stimulating the conversion of GTP to cGMP, while the indirect effect is through oxidation and nitration processes that can lead to altered protein structure and function.

Authors conclude that oxidative stress plays a significant role in most of the molecular and biochemical pathways in the cervix of the pregnant woman, and that complete understanding of the entire cervical ripening process seems crucial in developing new effective methods to cope with adverse pregnancy outcomes such as preterm labour.

Didziokaite G. et al. [3] present connection between oxidative stress, endometriosis, and infertility. There are several identified mechanisms by which oxidative stress can affect women’s fertility. ROS affect a number of physiological and pathological activities in the ovary and in the peritoneal environment. A certain amount of ROS is required to induce ovulation. Oxidative stress is the main mechanism by which the oocyte loses its developmental capacity after ovulation. Diminished ovarian reserve seems to be associated with oxidative stress and describes a decline in the number of oocytes in the ovary, which results in reduced female fertility and abnormalities of the reproductive endocrine system. A number of oxidative stress biomarkers collected from different sites such as serum, peritoneal fluid, follicular fluid, ovarian cortex, eutopic, and ectopic endometrial tissues of women diagnosed with endometriosis were examined in different studies. There are several mechanisms identified which support the theory that endometriosis may be caused by oxidative stress itself.

The authors conclude that endometriosis is one of the most common infertility-associated diseases and is suggested to be one of the causes of increased oxidative stress, and that there is evidence that the increased ROS production is the cause of endometriosis development as well as co-existing oxidative-stress-related infertility. This theory could explain the underlying OS-related cause of infertility in cases when only minimal or mild endometriosis is diagnosed.

Jovandarić ZM et al. [4] in their review article highlight that pregnant women and children can also become infected with SARS-CoV-2 virus, although more often they are only the carriers of the virus. Infant scan develops a severe form of the disease with a fatal outcome.

Placental blood vessels, as well as the placental tissue itself, are similar to the lung tissue, and the SARS-CoV-2 virus S protein binds to the placental blood vessels via the ACE2 receptor, causing the viral genomic RNA to be inserted into a host cell. membranes, causing the viral receptor causes a downregulation of this receptor, disrupting its normal function in maintaining immune homeostasis and leading to pro-inflammatory effects that can cause lung injury. The respiratory distress syndrome in a pregnant woman can affect the supply of oxygen to the foetus and initiate the mechanism of metabolic disorders of the foetus and new-borns, which are caused by asphyxia. As a consequence, proteins, DNA, lipids and other biomolecules are damaged, which leads to pathological changes in the body.

In the last part of the article, authors present COVID-19 disease in new-borns. They point out that mitochondrial damage is a central event in a cell that is affected by ischemia as a consequence of asphyxia in severe respiratory distress caused by a COVID-19 infection in a new-born infant. Additionally, a kind of programmed cell death occurs which is characterized by an imbalance in intracellular iron metabolism or a distortion of the glutathione peroxidation pathway. The transferrin receptor recognizes excess transferrin carrying Fe3+, and it enters cells through endocytosis after a SARS-CoV-2 infection.

Authors conclude that COVID-19 is not only an infectious disease caused by the SARS-CoV-2 virus, but it is also a metabolic disease. Lipid peroxidation mediated by asphyxia caused by the COVID-19 disease can cause the death of a new-born and pregnant women as well as short-term and long-term sequelae.

Komsa-Penkova R. et al. [5] in their article presented results of an altered composition or intermolecular interactions of the plasma proteome of women with early pregnancy loss (EPL). The embryo develops in a low-oxygen environment, thus protecting differentiating cells from damaging reactive oxygen species (ROS). An over-expressed inflammatory reaction and ROS production can lead to pregnancy complications or early miscarriage. The discovery of new biomarkers in biological fluids is one of the still unresolved challenges that researchers are facing due to the high molecular components’ complexity, as well as their wide and dynamic concentration range in the bloodstream. The dramatic changes that occur in the mother’s body also exert an effect on the blood plasma proteome from the beginning of pregnancy to birth. Blood plasma is a potential source of protein biomarkers for the discovery of many pathologies. Most of the studies are focused on the presence, absence, or relative abundance of specific proteins, and deviations in their molecular interactions. Differential scanning calorimetry (DSC) is used intensively to characterize the thermodynamic behaviour of blood plasma/serum proteome in health and disease. In their study, the DSC approach is used to study the denaturation profiles of blood plasma derived from EPL patients.

The obtained results clearly demonstrate the altered thermal behaviour of the plasma proteome of women with EPL (58% of cases). Thermal stabilization of a fraction of albumin assigned transition with concomitant suppression of the major and enhancement of the globulin-assigned transition are characteristic features of EPL DSC profiles, that could be used as a new indicator of a risk pregnancy. The presented results suggest altered composition or intermolecular interactions of the plasma proteome for EPL women.

McCracken SA et al. [6] examined the factors driving the hypoxic response in severely preterm PE and IUGR placentae compared to the spontaneous preterm (SPT) controls using immunoblotting, RT-PCR, immunohistochemistry, proximity ligation assays, and Co-IP.

To determine whether the pathological mechanisms that underlie PE and idiopathic IUGR share a molecular signature, they compared the oxygen sensing/response pathways in the placentae from early-onset PE and severe IUGR with gestationally age-matched controls. They found HIF-1α and -2α, but not HIF-1β, expression was elevated in severe IUGR and early-onset PE, and disproportionately increased in the syncytiotrophoblast layer. The HIF-α-subunits were transcriptionally active as the level of target gene sFlt-1 was enhanced in both pathologies while the association of VHL and HIF-1α was impaired in pathological pregnancies. Their study demonstrates that HIF-1α and HIF-2α protein expression is elevated at the post-transcriptional level in the placentae of normotensive women with severe IUGR and women with early-onset PE.

Authors conclude that because of the heterogenicity associated with pathological placentation, it is unlikely that one underlying mechanism drives all IUGR and/or PE. Rather, there are likely to be multiple mechanisms that exist that may be specific to the phenotype associated with the pathology.

Abedin Y. et al. [7] in their study determined the expression of Notch family genes and proteins and investigated the therapeutic effect of γ-secretase inhibitors (GSIs), indirect inhibitors of Notch signalling, in uLMS. They determined the expression of Notch genes and proteins in benign uterine smooth muscle tissue, fibroids, and uLMS samples by immunostaining and in two uLMS cell lines, SK-UT-1B (uterine primary) and SK-LMS-1 (vulvar metastasis) by RT-PCR, Western blot and immunostaining. They exposed their cell lines to GSIs, DAPT and MK-0752, and measured the expression of HES1, a downstream effector of Notch.

From the presented results, we see that Notch signalling is active in uLMS cell lines and that inhibition of γ-secretase with both DAPT and MK-0752 inhibits Notch signalling and decreases uLMS cell viability. This is the first study that determined Notch expression in uLMS tissue samples and showed that Notch signalling is active in uLMS, making the Notch signalling pathway a potential therapeutic target. Their study is unique in that we identified a potential targetable pathway for this rare gynaecologic malignancy.

Authors conclude that Notch signalling was active, and Notch genes and proteins were differentially expressed in uLMS. GSIs decreased Notch signalling in uLMS in a dose- and time-dependent manner. Taken together, these findings have identified the Notch signalling pathway as a potential therapeutic target for uLMS.

Shvestova AA et al. [8] tested the hypothesis that, in early ontogenesis, the action of highly abundant NO is favourable for the expression of genes specific to the contractile phenotype of arterial smooth muscle. If so, NO deficiency during intrauterine development would attenuate the expression of smooth muscle differentiation markers. To test this, they supplemented pregnant rat females with NOS inhibitor, to suppress NO production in developing foetuses.

The results show that endothelium-dependent relaxation of the aorta to acetylcholine was decreased in new-born pups from the L-NAME group due to the reduction of NO-mediated signalling, as was shown using an inhibitor L-NNA. Smooth muscle responsiveness to NO did not differ between groups because the reduction in NO-mediated signalling in pups born to L-NAME-treated females had an endothelial origin. Aortic smooth muscle cells of new-born rat pups from the L-NAME group are less differentiated because of NO deficiency during development.

Authors conclude that their results based on an in vivo model of maternal NO-deficiency demonstrate that NO is important for smooth muscle cell-specific gene expression during early ontogenesis. A high level of NO production in early ontogenesis is important not only for maintaining a low level of blood pressure in still-immature vessels but is also necessary for phenotypic modulation of vascular smooth muscle cells, their transition to a mature contractile phenotype.

This Special Issue brings together highly scientific articles in which the authors present their own research or a journal account of how pregnancy and various pathologies are linked to oxidative stress. Each contribution is an important piece in the mosaic of knowledge and understanding of the mechanisms that may lead to premature birth or to growth restriction or even to the death of the mother or the new-born. However, given that we are still in the COVID-19 era, it is important to understand the long-term impact of infection in pregnant women.
